# Portable Point-of-Care Diagnosis Platforms and Emerging Predictive Biomarkers for Rapid Detection of Severe Dengue Viral Infection

**DOI:** 10.1021/acssensors.5c00263

**Published:** 2025-04-01

**Authors:** Tharmaraj Vairaperumal, Po-Tseng Lee, Ping-Yen Liu

**Affiliations:** † Institute of Clinical Medicine, College of Medicine, 38026 National Cheng Kung University, Tainan 70403, Taiwan, ROC; ‡ Division of Cardiology, Department of Internal Medicine, National Cheng Kung University Hospital, College of Medicine, National Cheng Kung University, Tainan 70403, Taiwan, ROC

**Keywords:** Dengue diagnosis, Dengue virus, DENV, point-of-care, predictive biomarkers, portable device, rapid detection, viral infection

## Abstract

Dengue virus (DENV) infection is a major global public health problem, particularly in tropical and subtropical regions where Aedes mosquitoes are prevalent. The clinical spectrum of dengue ranges from mild febrile illness to severe conditions such as dengue hemorrhagic fever and dengue shock syndrome. Early prediction of dengue progress is crucial for timely therapeutic medications, which can reduce both morbidity and mortality. Traditional diagnostic methods such as serological tests and polymerase chain reactions are often time-consuming and require sophisticated infrastructure and skilled personnel. To overcome these limitations, the development of point-of-care (POC) diagnosis platforms and novel predictive biomarkers is crucial to providing rapid, real-time diagnostic tools that can be used in low-resource settings and at the patient’s bedside. Predictive biomarkers enable the identification of disease risk in the early stages and can reduce hospitalization visits. This review offers a comprehensive overview of portable POC diagnosis platforms and emerging predictive biomarkers for the rapid diagnosis of severe DENV infection. Its provides an overview of its epidemiology, discusses the global burden of DENV, and explores DENV infection with different serotypes, as well as the clinical spectrum and severity of dengue. The key focus is on the latest advancements in POC diagnosis readout methods and portable POC devices for DENV diagnosis, including colorimetric assay, electrochemical method, lateral flow strip, and microfluidic chip platforms. In addition, the review article explores various emerging predictive biomarkers for the rapid detection of DENV, while also highlighting the limitations associated with protein, nucleic acid, and metabolic biomarkers. Finally, we address the current challenges, limitations, and potential future directions of POC diagnosis platforms for the diagnosis of severe DENV infection.

## Graphical abstract

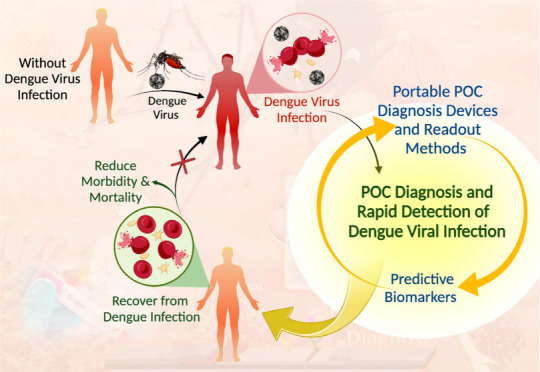

Dengue virus infection is an important global public health concern, particularly in tropical and subtropical regions where Aedes mosquitoes are the primary vectors.^1^ Dengue has significantly affected more than 100 countries in Southeast Asia, and the burden of the disease in five key nations, including Indonesia, Thailand, Malaysia, the Philippines, and Vietnam, was estimated from outbreak data over the past three decades.^2^ An in-hospital mortality rate of 1.34% was recorded during the 2015 dengue outbreak in Tainan, Taiwan, with 60 deaths among 4,488 patients admitted for treatment.^3^ These data underscore the ongoing risk of dengue epidemics and sporadic outbreaks in the near future.

The clinical manifestations of dengue range from mild, self-limiting febrile illness to severe, life-threatening conditions such as dengue hemorrhagic fever (DHF) and dengue shock syndrome (DSS).^4^ Predicting the progression of mild to severe disease forms is crucial for the timely implementation of therapeutic interventions, which can significantly reduce both morbidity and mortality. Traditional diagnostic methods, such as serological tests and polymerase chain reaction (PCR), are essential for the confirmation of dengue infection.^5,6^ However, these methods often require a sophisticated laboratory infrastructure, are time-consuming, and depend on skilled personnel to provide accurate results. In addition, clinical similarity with other febrile diseases, cocirculation of multiple serotypes (DENV-1 to DENV-4),^7^ and the phenomenon of antibody-dependent enhancement (ADE),^8^ which complicates disease progression and diagnosis. To address these limitations, there is a crucial need for rapid, real-time, on-the-spot diagnostic tools that can be used at the patient’s bedside or in primary care settings.

Point-of-care diagnostics has emerged as a crucial innovation for the diagnosis and management of various infectious diseases, particularly dengue.^9^ POC-based biosensor devices and novel predictive biomarkers have shown immense potential in the diagnosis of infectious diseases.^10^ Interestingly, POC diagnostic platforms have been successfully used to detect viral diseases such as Zika and Chikungunya, which share clinical and epidemiological similarities with dengue.^11^ These advances underscore the versatility of POC diagnosis systems in addressing the public health challenges posed by arboviral infections. POC diagnosis has emerged as a crucial innovation in the diagnosis and management of infectious diseases.^10^ POC diagnosis platforms are designed to be rapid, easy to use, and require minimal equipment, making them particularly suitable for low-resource settings. Recent advances have focused on identifying biomarkers and risk factors that predict the outcomes of severe dengue.^12^ POC technologies have helped identify novel biomarkers to predict severe dengue infection, such as viral proteins such as nonstructural protein-1 (NS1), endothelial activation markers, and host-derived genetic biomarkers. These include platelet indices, cytokines, and other inflammatory mediators, which play a crucial role in determining the severity of the disease.^13^

Recent review articles have explored various POC diagnostic modalities for infectious diseases, including HIV, tuberculosis, influenza, and dengue.^14,15^ This review emphasizes the genetic diversity and cocirculation of dengue virus serotypes (DENV-1 to DENV-4), overlap of symptoms with other febrile illnesses, and the critical need for early diagnosis due to rapid disease progression. We also highlight recent technological advancements in portable POC readout methods and devices, including colorimetric assay, lateral flow strip, microfluidic chip, and electrochemical platform for DENV diagnosis. Furthermore, this review explores various novel predictive biomarkers for the rapid detection of DENV. Additionally, we discuss current challenges, limitations, and future directions for PoCT platforms in DENV infection management. Figure 1 presents a schematic illustration representing DENV infection, associated symptoms, portable POC diagnosis platforms, and predictive biomarkers for the rapid detection of DENV.

**Figure 1:**
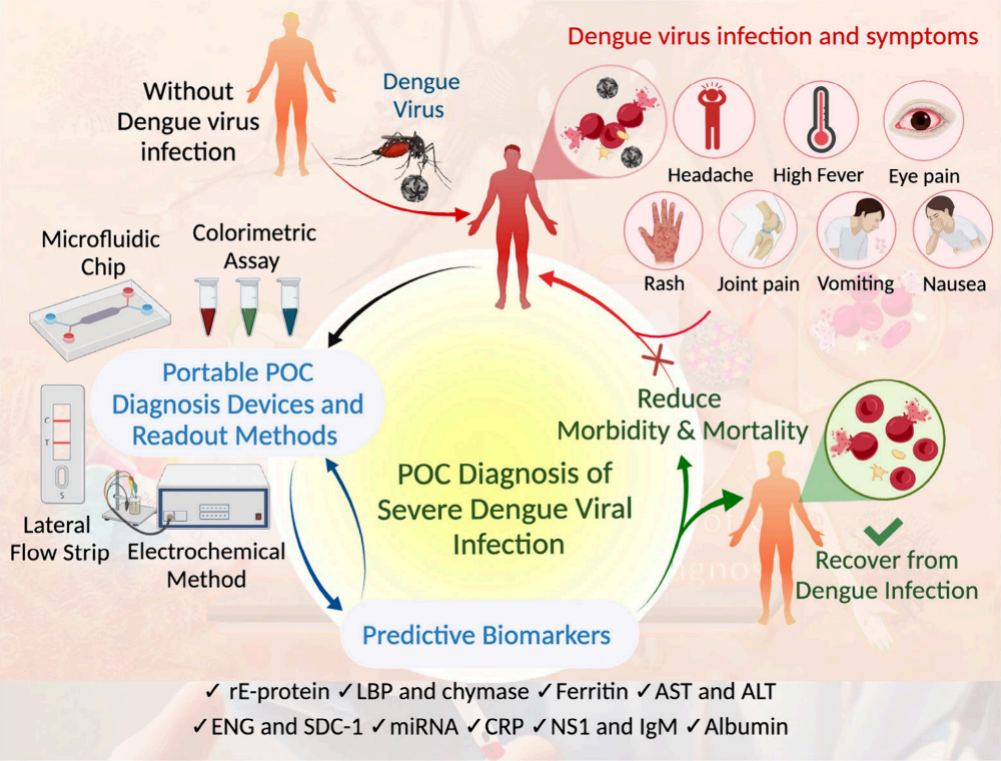
Schematic illustration of symptoms associated with DENV infection, portable POC diagnosis platforms, and emerging predictive biomarkers for the rapid detection of DENV.

## Overview of DENV and Disease Mechanisms

### Epidemiology and Global Burden of Dengue

Dengue fever is a mosquito-borne disease that has become a major global threat to public health and human life. Over the past 50 years, the incidence of dengue fever has increased dramatically, with approximately half of the world population now at risk. Dengue fever is endemic in more than 100 countries, and the World Health Organization (WHO) estimates that it affects approximately 2.5 billion people around the world.^16^ The disease is particularly prevalent in regions such as Asia, Africa, South America, and the Caribbean, with an estimated 390 million cases per year.^17^ In certain areas, severe clinical manifestations of dengue can result in mortality rates ranging from 5% to 20%.^18^ The global spread of dengue fever is worsened by the lack of effective antiviral treatments and vaccines, along with factors such as climate change, international travel, and rapid urbanization, making it a significant public health challenge.

### DENV Infection and Its Serotypes

DENV is classified within the Flaviviridae family and the Flavivirus genus.^19^ It exists in four distinct serotypes (DENV 1–4) that affect humans, with a fifth serotype (DENV5), first identified in 2007, limited to the sylvatic cycle.^20^ The DENV genome is composed of single-stranded RNA that encodes 10 proteins: three structural proteins such as membrane (M), envelope (E) and capsid (C) that contribute to the architecture of the virus, and seven nonstructural proteins (NS1, NS2A, NS2B, NS3, NS4A, NS4B, and NS5) that play crucial roles in RNA replication.^21^

### Clinical Spectrum and Severity of DENV

The clinical spectrum of DENV infection ranges from mild dengue fever (DF) to severe dengue, characterized by dengue hemorrhagic fever or dengue shock syndrome.^22^ Dengue fever typically manifests itself as an acute febrile disease characterized by severe headache, retroorbital pain, myalgia, and arthralgia, often referred to as ″break-bone fever″.^23^ In contrast, severe dengue manifests itself as a more serious clinical syndrome, marked by vascular leakage that leads to plasma loss, which can result in hypovolemic shock and DSS.^24^ Furthermore, DHF is characterized by hemorrhagic manifestations, including petechia, bleeding gums, and, in severe cases, gastrointestinal bleeding.^25^ The progression from mild dengue fever to severe dengue, including manifestations such as vascular leakage and plasma loss, underscores the critical need for early detection and intervention. These clinical characteristics emphasize the role of predictive biomarkers and POC diagnosis tools in stratifying disease severity and optimizing patient care.^26^

## Rapid Diagnosis of Severe DENV Infection

The diagnosis of dengue fever depends on a combination of clinical evaluation and laboratory tests. Traditional diagnostic approaches primarily include serological assays to detect dengue-specific antibodies (IgM and IgG) or viral RNA through polymerase chain reaction (PCR).^27^ IgM antibodies are typically detectable between days 3 and 5 after infection, whereas IgG antibodies appear later and can persist for months to years. Thus, serological testing is valuable for distinguishing primary from secondary dengue infections.^28^ However, these antibody-based methods are subject to diagnostic limitations, particularly in the early stages of infection, where the host’s immune response may not yet be fully developed, leading to false negatives.

In antigen-based immunoassays, such as those targeting nonstructural protein 1 (NS1), enable earlier detection of dengue virus. NS1 antigen is present in the bloodstream on day 1 after infection, making it a crucial biomarker for early diagnosis.^29,30^ Notably, NS1-based detection methods achieve limits of detection as low as 1.63 pg/mL, while serological assays typically exhibit thresholds in the range of 1–10 ng/mL.^5,31^ By integrating antigen-targeting immunoassays with POC diagnostic platforms, rapid and accurate dengue detection can be achieved, particularly in resource-limited settings where early disease identification is critical for effective management. Such advances facilitate timely clinical interventions, reducing the risk of severe complications, and improving patient outcomes. Therefore, the development and implementation of highly sensitive and selective POC diagnostic tools are essential to identify people at risk of severe dengue and optimize disease management strategies.

## POC Diagnosis Platforms for DENV

Portable POC diagnosis devices and readout methods are crucial to improve patient care and treatment outcomes.^32^ These technologies provide rapid results at the patient’s bedside, significantly reducing the time between diagnosis and the initiation of treatment. Figure 2 presents a schematic illustration of the clinical workflow and highlights the key advantages of using POC diagnosis platforms in healthcare settings. This section explores the recent development of portable POC diagnosis devices and readout methods, including colorimetric assay, electrochemical method, lateral flow strip, and microfluidic chip platforms with the potential to improve the efficiency of the diagnosis of severe dengue.

**Figure 2:**
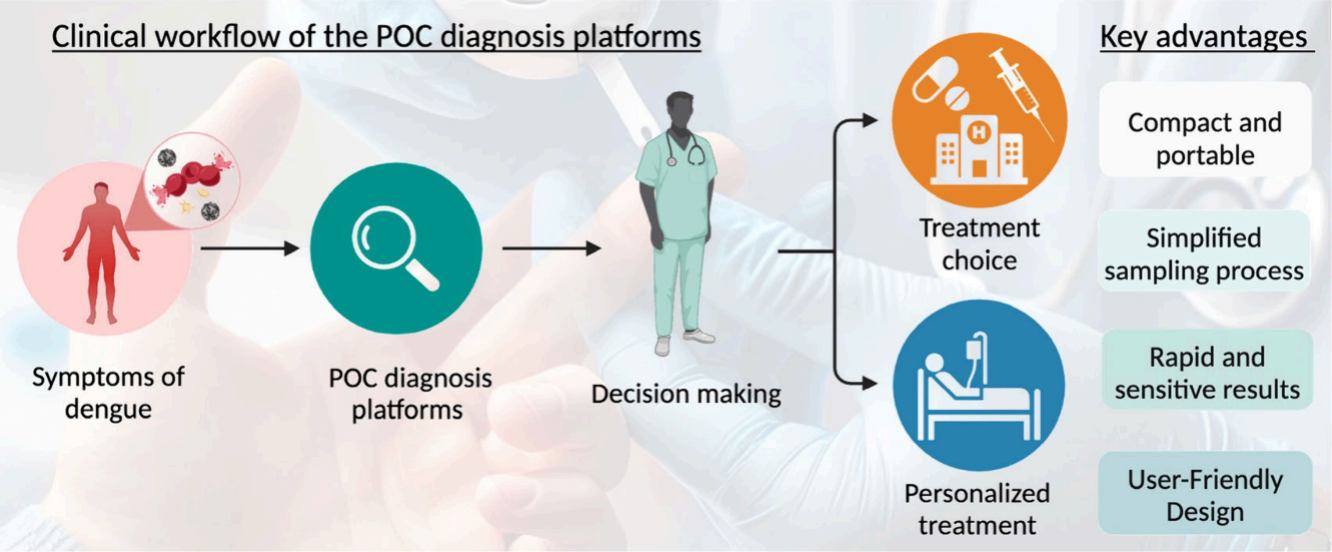
Schematic illustration of the clinical workflow and highlighting the key advantages of POC diagnosis platforms that provide rapid results at the patient’s bedside.

### POC Readout Techniques

#### Colorimetric Assays-Based Detection of DENV

Colorimetric assays are widely recognized for their simplicity and ease of use on POC diagnosis platforms. Colorimetric assays detect the presence of specific analytes through a color change in the reagent, which can be observed with the naked eye or measured using simple portable devices.^33,34^ In the context of the diagnosis of severe dengue, colorimetric assays offer rapid and straightforward detection of viral antigens or antibodies. In particular, the Aptamer-associated reverse transcription loop-mediated isothermal amplification (APTA-RT-LAMP) colorimetric assay demonstrates a sensitivity of 95% and a specificity of 100% for detecting DENV with the naked eye.^35^ Similarly, an RT-LAMP method based on 3′-NCR gene sequences has been developed for the naked eye colorimetric detection of DENV 1–4 in 30–45 min and to achieve 100% sensitivity.^36^ Recently, acid-controlled aggregation of DNA-functionalized plasmonic AuNPs has been employed for the highly sensitive colorimetric detection of DENV.^37^ DNA-functionalized triangular AuNPs hybridize with the genomic RNA of the virus, forming RNA-DNA heteroduplexes. This hybridization leads to nanoparticle aggregation, altering the localized surface plasmon resonance (LSPR), and resulting in a visible color change. The color transition occurs because of changes in interparticle spacing, which modulate the optical absorption properties of the nanoparticles. This approach enables the sensitive detection of DENV with a detection limit as low as ∼ 1 pg/μL, as determined by simple spectroscopic analysis. Another development involves magnetic immuno-nanoplatforms for specific colorimetric detection of the NS1 protein in serum samples, using Fe_3_O_4_ NPs conjugated with anti-NS1 antibodies (Ab42316).^38^ This platform produces a visible blue color change, detectable by the naked eye, within a concentration range of 0.415 to 1.24 μg/mL.

Plasmonic magnetic Fe_3_O_4_/Au shell nanoparticles (PMNs) have also been used, and the timeline with the workflow of the developed plasmonic photothermal reverse transcription colorimetric polymerase chain reaction platform (PPT-RTcPCR) for DENV detection has also been used, as shown in Figure 3a.^39^ The PPT-RTcPCR method takes advantage of plasmonic photothermal effects, where Fe_3_O_4_/Au nanoparticles absorb specific wavelengths of light, generating localized plasmonic photothermal effects. These localized plasmonic photothermal effects accelerate the reverse transcription and polymerase chain reaction cycles, significantly reducing the assay time. Furthermore, the enhanced signal amplification provided by the nanoparticles contributes to the assay, which achieved a remarkably low limit of detection of 1.6 copies/μL with exceptional sensitivity (97%) and specificity (100%). Similarly, surface-active Fe_2_O_3_@AuNPs conjugated with aptamers exhibited highly specific and selective colorimetric real-time detection of the four dengue serotypes.^40^ The Aptamer-Fe_2_O_3_@AuNPs colorimetric assay tests provide the results by quickly visually indicating color changes upon detection of DENV antigens or antibodies. Additionally, poly­(A) ssDNA functionalized with triangular silver nanoparticles (TAg NPs) has been utilized for serotype-specific colorimetric detection of DENV RNA.^41^ The TAg-DNA probe forms an aggregated network-like structure specifically with the target RNA, and this process stabilizes in the presence of NaCl, which is presented in Figure 3b.

**Figure 3:**
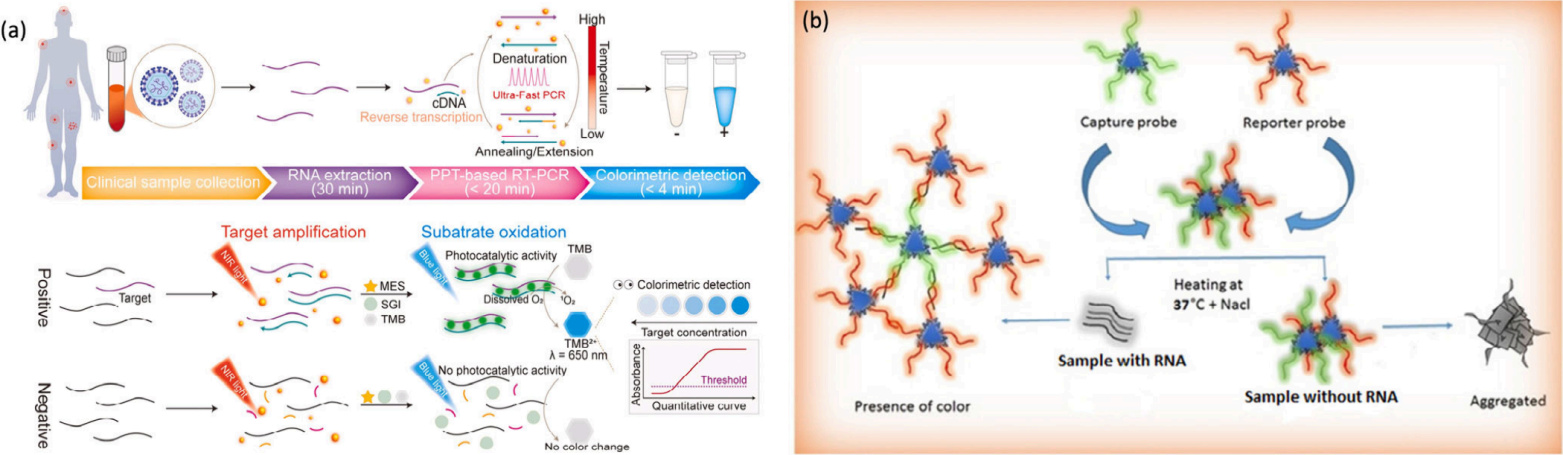
(a) Workflow of the plasmonic magnetic Fe_3_O_4_/Au shell NPs-based colorimetric readout platform PPT-RTcPCR for DENV detection. Adapted with permission from ref.^39^ Copyright 2023 Permission from Elsevier. (b) DNA-functionalized triangular AgNPs probe for serotype-specific colorimetric detection of DENV RNA in the presence of NaCl. Adapted with permission from ref.^41^ Copyright 2018 Permission from Elsevier.

Colorimetric assays stand out for their visual simplicity, allowing rapid detection of DENV biomarkers through observable color changes without the need for sophisticated instruments. Although plasmonic nanoparticle-based colorimetric assays exhibit high sensitivity and specificity, they may encounter potential limitations in real-world applications.^33^ Environmental factors such as temperature fluctuations, humidity, and prolonged storage can affect nanoparticle stability and assay performance.^42^ For instance, aggregation or degradation of nanoparticles under insignificant conditions could lead to false positives or reduced sensitivity. Additionally, nonspecific binding events in complex biological samples may contribute to false negatives or positives.^43^ Future developments should focus on optimizing nanoparticle formulations and incorporating stabilizing agents to enhance the assay robustness in various operational settings.

#### Electrochemical-Based POC Detection of DENV

Electrochemical sensors operate by measuring electrical signals generated during redox reactions at the electrode surface, with analyte interaction facilitating signal transduction.^44^ These platforms provide high sensitivity, which is critical for detecting low-abundance dengue biomarkers. Furthermore, electrochemical sensors can be integrated into portable devices, enabling real-time monitoring and rapid quantification of viral loads.^45^ Recently, numerous innovative approaches for electrochemical-based POC diagnostic platforms for severe dengue infection have emerged in the literature. In particular, the first development of a label-free electrochemical capacitive POC method to detect the NS1 DENV biomarker in human serum samples.^46^ Figure 4a illustrates the design and integration of capacitive sensing mechanisms in a label-free capacitive POC diagnostic platform, optimized for the rapid and sensitive detection of the biomarker of NS1 DENV. This method used a modified surface of ferrocene-tagged peptides integrated with an anti-NS1 capacitive assay, demonstrating statistically significant performance with a p-value of <0.01 and a cutoff value of 1.36%. In another approach, a differential pulse voltammetric (DPV) electrochemical biosensor platform was developed for DENV-NS1 detection using a bioconjugated anti-NS1 antibody and a screen-printed gold electrode modified with polydopamine (PDA).^31^ This biosensor exhibited a low LOD of 1.63 pg/mL and a wide linear detection range from 10 pg/mL to 1 ng/mL (R^2^ ∼ 0.969), alongside acceptable sensitivity, specificity, and precision of sensitivity, specificity, and precision of 90.00%, 80.95%, and 87.65%, respectively. Similarly, carbon nanotubes covalently immobilized with anti-NS1 monoclonal antibodies were used in a DPV electrochemical screening immunosensor for the detection of dengue and Zika viruses.^47^ This immunosensor showed a linear range of 20 to 800 ng/mL and a reproducibility of 3.0%, with an LOD as low as 6.8 ng/mL in spiked urine and real serum samples.

**Figure 4:**
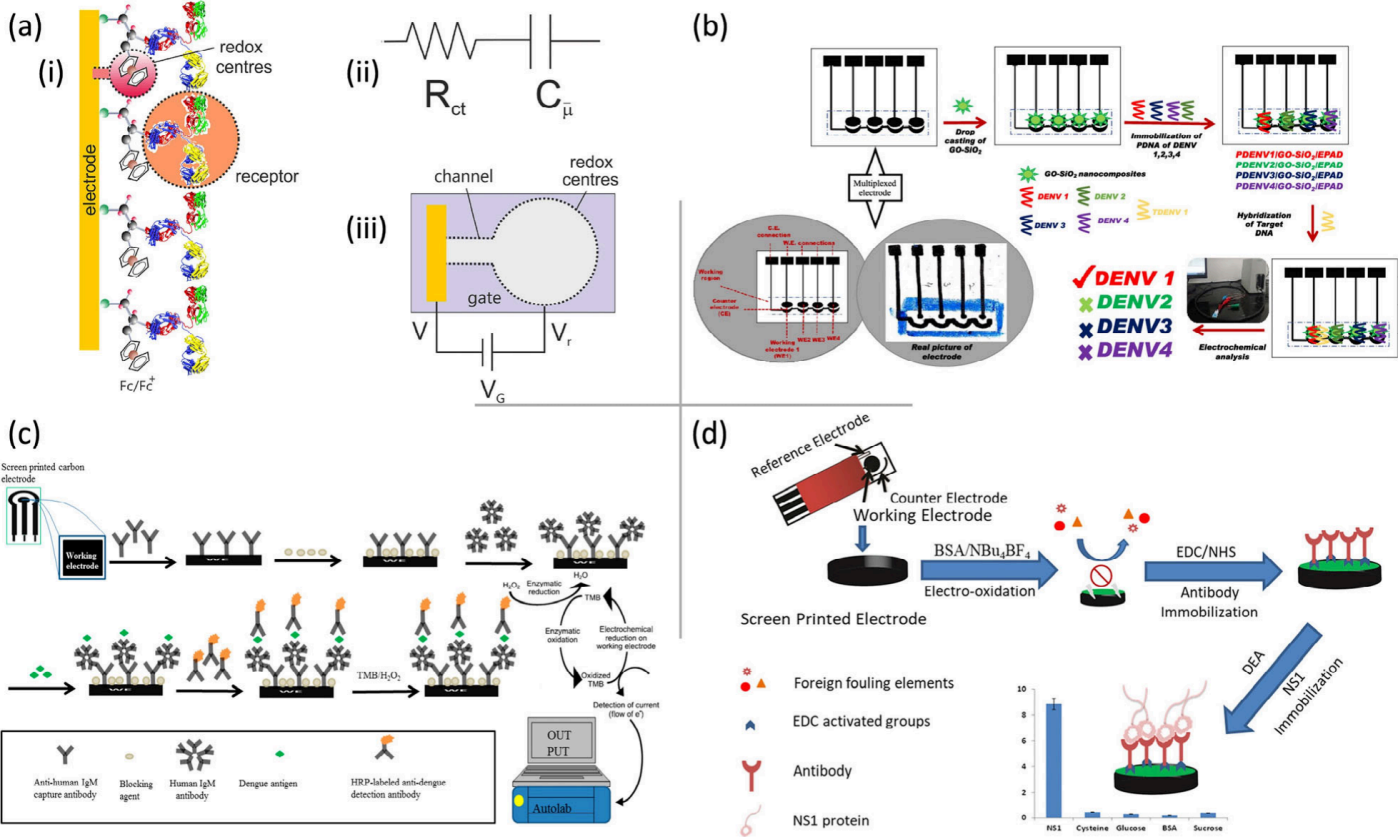
(a) Schematic representation of a label-free capacitive PoCT diagnostic platform: (i) a redox-active monolayer at the biological receptor interface, (ii) the corresponding electrochemical capacitive circuit, and (iii) the capacitive moieties unit. Adapted with permission from ref.^46^ Copyright 2020 Permission from Elsevier. (b) Design of a paper-based multiplexed platform with parallel electrodes for the detection of all four DENV serotypes. Adapted with permission from ref.^49^ Copyright 2020 Permission from the American Chemical Society. (c) Schematic diagram illustrating the design and workflow of the SCPE-based dengue IgM biosensor platform. Adapted with permission from ref.^54^ MDPI 2021 Copyright Permission. (d) Fabrication of the SCPE-based impedimetric immunosensor platform and modification of the working electrode for ultrasensitive detection of DENV. Adapted with permission from ref.^55^ Copyright 2018 Permission from Elsevier.

Furthermore, a label-free DNA hybrid-based POC electrochemical biosensor platform was developed using chiral-induced spin selectivity (CISS) for the detection of DENV, achieving an LOD as low as 0.12 pM.^48^ Another innovative platform, shown in Figure 4b, is a paper-based multiplexed electrochemical nanosensor designed for the simultaneous detection of the four DENV serotypes.^49^ This developed multiplexed electrochemical genosensor, using graphene oxide-silicon dioxide (GO-SiO_2_) nanocomposites, was engineered to detect DENV-1, DENV-2, DENV-3, and DENV-4 in a broad concentration range, 100 pM to 100 μM. Another approach, a single-chip disposable multiplex POC electrochemical platform, was also developed using a gold surface functionalized with cysteamine and antibodies against NS1 proteins for the detection of Zika and Dengue.^50^ This platform exhibited a strong linear correlation between the percentage change in impedance and the logarithm of the NS1 concentration, with R^2^ values of 0.990 for Dengue and 0.995 for Zika.

The development of a highly sensitive polyethylenimine-modified high-surface-area porous carbon-based POC diagnostic platform has enabled early detection of DENV.^51^ This glassy carbon electrode platform (GCE) demonstrated high sensitivity, with a linear detection range of 1 pg/mL to 100 μg/mL and a LOD of as low as 0.665 pg/mL. In addition, screen-printed electrodes (SPEs) are widely utilized in the development of POC electrochemical biosensor platforms, because of their many advantages, including cost effectiveness, simplicity, scalability, versatility, portability, and compact size.^52^ Recently, a screen printed carbon electrode (SPCE)-based electrochemical biosensor modified with 1-ethyl-3-(3-(dimethylamino)­propyl) carbiimidehydrochloride/*N*-hydroxysulfosuccinimide (EDC s-NHS) was developed for the rapid detection of DENV in human plasma, achieving detection limits as low as 0.16 nM in 5 min.^53^ Similarly, goat antihuman IgM antibody immobilized SPCEs (GAHICA) were developed for the detection of dengue-specific IgM antibodies in serum samples, as shown in Figure 4c. The GAHICA immobilized SPCE platform demonstrates 100% sensitivity and specificity, with an LOD of 1:16% serum dilution.^54^ Furthermore, bovine serum albumin (BSA) was designed as a POC electrochemical immunosensor platform for the early diagnosis of DENV.^55^ The fabrication process and the workflow of the ultrasensitive impedimetric immunosensor platform based on SPCE modified with BSA are shown in Figure 6d. This portable SPCE immunosensor platform exhibited an enhanced LOD of as low as 0.3 ng/mL and a linear range of 1 to 200 ng/mL. These developed electrochemical platforms offer high sensitivity and specificity; these devices detect electroactive dengue biomarkers in real time. These POC diagnostic technologies have been instrumental in improving dengue diagnosis efficiency, significantly reducing the time from the onset of symptoms to treatment, and improving patient outcomes in clinical settings, particularly in resource-limited environments.

### Portable POC Diagnosis Devices

#### Lateral Flow Stripe-Based POC Detection of DENV

Lateral flow assays (LFAs) are widely recognized for their simplicity, rapid operation, and ability to produce results that are easily interpretable even by nonexperts. LFAs utilize solid-phase test strips for detection, distinguishing them from colorimetric assays, which are typically performed in solution.^56^ These features make LFAs ideal for POC diagnostics, particularly in resource-limited settings.^57^ Antibody-conjugated gold nanospheres (AuNSP@mAb)-based LFAs detect DENV NS1 antigen through antibody-conjugated test strips, whereas colorimetric assays involve solution-based optical changes driven by nanoparticle aggregation.^58^ This advanced thermal sensing assay can detect as low as 1.56 ng/mL, which is four times more sensitive than the typical visual readout of 6.25 ng/mL. Similarly, a DNA capture probe immobilized in dextrin-capped AuNPs was used to develop a colorimetric lateral flow biosensor (LFB) for the detection of dengue-1 RNA in pooled human sera, with a cutoff value of 1.2 × 10^4^ pfu/mL.^59^ An antibody (4G2)-conjugated AuNPs-based LFA has been reported for DENV detection, with a detection limit of 5.12 × 10^2^ PFU.^60^ Figure 5a illustrates the design of a rapid conjugate-based AuNPs@4G2 biosensor and an LFA test strip for flavivirus detection. In the test zone, DENV is detected, whereas the control zone contains a secondary antibody against IgG, leading to the generation of two signals, indicating a negative result. When interaction with a sample containing DENV, the AuNPs@4G2-DENV complex forms, generating a positive signal. For example, a DNA-conjugated AuNP-based POC rapid diagnostic test (RDT) using lateral flow biosensors (LFBs) has been developed for the detection of hemorrhagic fever viruses, achieving a detection limit as low as 10 fM.^61^ Although their simplicity and rapidity are notable, LFAs often face sensitivity challenges, which limits their ability to detect low-concentration biomarkers.

**Figure 5:**
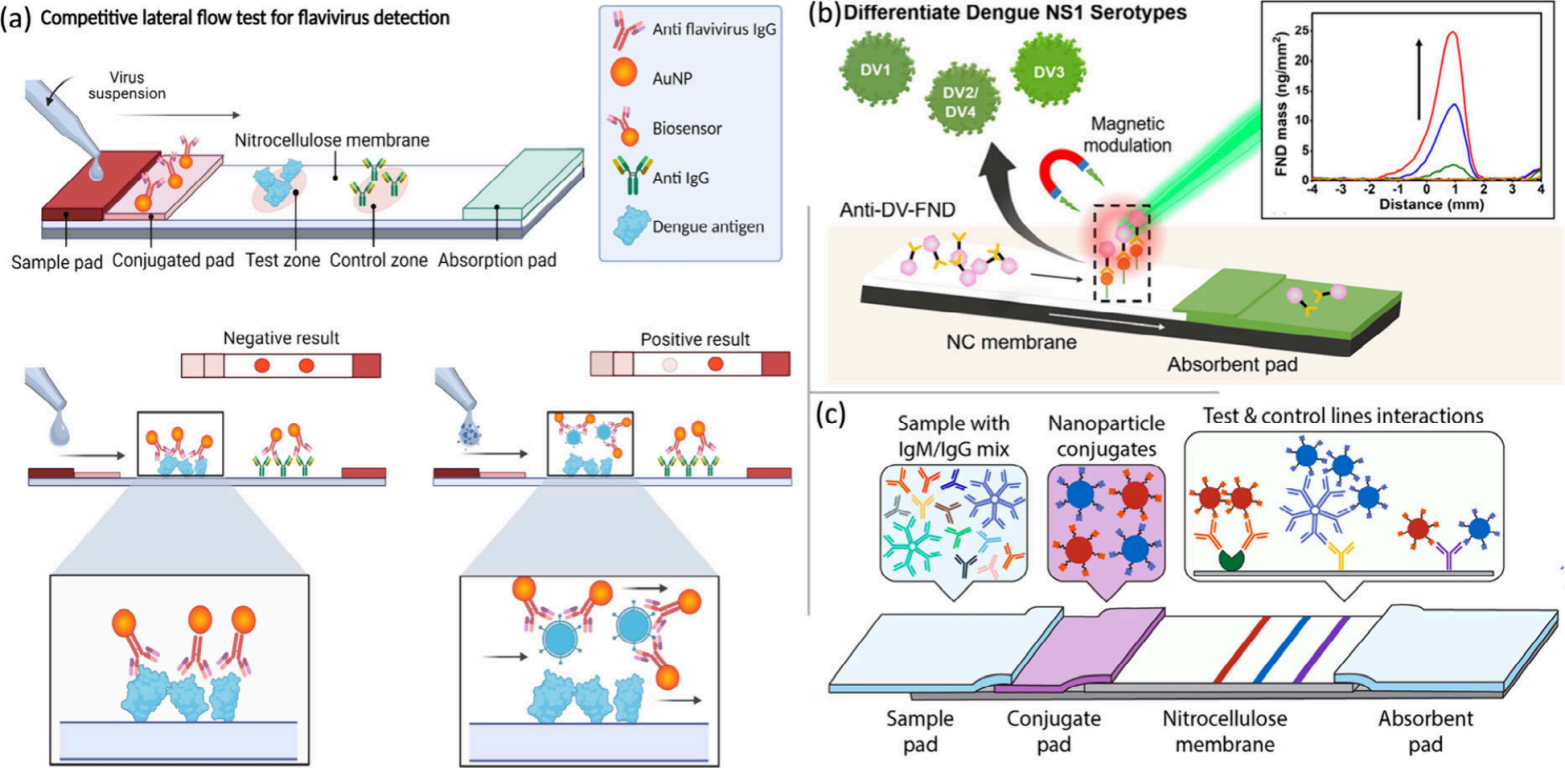
(a) Design and working principles of a rapid conjugate-based AuNPs@4G2 biosensor and LFA test strip for flavivirus detection. The performance of the AuNPs@4G2-DENV complex forms a negative result and a positive signal. Adapted with permission from ref.^60^ MDPI 2022 Copyright Permission. (b) Fabrication and working performance of the one-step physical adsorption of anti-NS1 antibodies conjugated to the fluorescent nanodiamond (FND)-SELFIA strip for Dengue NS1 detection approaches and intensity of the prominent mass peaks of the FND (insert). Adapted with permission from ref.^62^ Copyright 2022 Permission from the American Chemical Society. (c) The components and color mixing strategy of the blue latex nanoparticle-based LFA test strip enable multiplexed detection of DENV. Adapted with permission from ref.^65^ Copyright 2019 Permission from the American Chemical Society.

Recent innovations, such as spin-enhanced lateral flow immunoassays (SELFIA), have addressed this limitation by significantly improving the detection limits. SELFIA uses an antidengue antibody conjugated fluorescent nanodiamond suspension, achieving a highly sensitive linear detection range from 0.1 to 1.3 ng/mL for DENV NS1 protein serotypes.^62^ The performance of SELFIA in detecting dengue NS1 serotypes is illustrated in Figure 5b. Another approach, the POC reverse transcription recombinase-aided amplification (RT-RAA)-based POC dipstick (LFD) assay was developed for the detection of DENV RNA in 247 clinical serum samples (P = 0.957).^63^ The RT-RAA-LFD assay identified 64 (25.9%) negative samples and 183 (74.1%) positive samples, which closely matched the results obtained by real-time RT-PCR, with 62 (25.1%) negative samples and 185 (74.9%) positive samples. Furthermore, a horseradish peroxidase (HRP)-based lateral flow immunoassay based on the NS1 antibody was successfully developed for the quantitative detection of the dengue NS1 antigen, demonstrating a linear detection range of 1 to 300 ng/mL, with a correlation coefficient (R^2^) of 0.9798.^64^

Multiplex detection of DENV and other arboviruses, such as chikungunya, is crucial in regions where cocirculation is common. Co-infections can complicate diagnosis and treatment decisions. Multiplex LFAs allow simultaneous identification of multiple pathogens, allowing clinicians to pinpoint the primary cause of symptoms and tailor treatment strategies, particularly in cases of dual infections. A multiplex lateral flow immunoassay (LFIA) utilizing colloidal gold conjugated anti-DENV NS1 antibodies has been developed for the detection of DENV NS1 serotypes.^66^ The assay demonstrated detection sensitivities of 90.0%, 88.24%, 82.61%, and 83.33% with specificities of 98.74%, 96.13%, 99.39%, and 97.04%, for serotypes D1, D2, D3, and D4, respectively. In another study, a single test strip lateral flow platform employs a unique color mix-ing strategy that allows multiplexed detection of DENV and chikungunya virus (CHIKV) IgM/IgG antibodies.^65^ Figure 5c presents the design and working principles of the blue latex nanoparticle-based LFA test strip used in a rapid diagnostic platform for the detection of faint traces of color differentiation of infections in human clinical samples in 30 min using 1 μL.

A smartphone-integrated disposable point-of-care LFA diagnostic kit was developed for the detection of the NS1 protein biomarker in clinical serum samples, using an AuNPs-conjugated antibody immunoprobe.^67^ The immunochromatographic strip exhibited a detection limit of 10 pg/mL, with a characteristic wine-red color change that indicates the presence of the target protein. Paper-based nucleic acid extraction and testing LFA platforms have been developed for the rapid detection of viral RNAs from spiked serum and complex clinical samples <1 h, with an LOD as low as a single copy in phosphate buffered saline (PBS) and 10 copies in serum.^68^ Similarly, a portable, wax-printed smartphone-integrated LFA paper-based analytical device (PAD) has been developed using a conjugated immunoassay of AuNPs anti-NS1 antibody (AuNPs @ anti-NS1) for colorimetric and quantitative detection of dengue NS1 antigen.^69^ This DEN-NS1-PAD demonstrated a remarkable LOD of as low as 200 ng/mL for visual detection and 112.19 ng/mL using the mobile app, producing qualitative color reads in 20–30 min. Furthermore, an automated smartphone-integrated microfluidic sandwich enzyme-linked assay-based POC device for DENV infection demonstrated remarkably high sensitivity, with a detection limit of 62.5 ng/mL in whole plasma.^70^ These developed lateral flow assays are user-friendly and produce rapid results.

Table 1 provides a comparison of different sensing material-based LFA platforms, highlighting their detection performance and practical characteristics such as the limit of detection (LOD), accuracy, cost-effectiveness, and multiplexing capabilities of the following sensing materials. AuNSP@mAb, DNA probes, and fluorescent nanodiamonds (FNDs) are considering the suitability of materials for specific DENV diagnostic applications. In addition, they compare their detection limits with standard diagnostic techniques such as ELISA and PCR. ELISA achieves a detection limit of ∼10–100 pg/mL, while PCR can detect RNA up to a few copies per reaction (∼10 fM). Although LFAs may not always match PCR sensitivity, their rapid results, portability, and lower cost make them invaluable in POC settings. Although LFAs demonstrate remarkable sensitivity and selectivity, their practicality in real-world applications depends on factors such as cost, stability, and shelf life. LFAs that incorporate gold nanoparticles are moderately priced but require careful handling to avoid degradation. Fluorescent nanodiamond-based LFAs, while highly sensitive, are costlier, potentially limiting their widespread adoption. Stability at extreme temperatures and prolonged storage conditions also influence its usability in resource-limited settings.^57^ Future designs should emphasize robustness and cost reduction to improve accessibility and reliability.

**Table 1:** Comparison of Different Sensor Material-Based LFA Platforms^a^

material	LOD	accuracy	cost-effectiveness	multiplexing capabilities	ref
AuNSP@mAb	1.56 ng/mL	high	moderate	limited	^58^
DNA probes	10 fM	very high	low	moderate	^61^
FND	0.1–1.3 ng/mL	very high	high	high	^62^

a AuNSP@mAb, Antibody-conjugated gold nanospheres; DNA, Deoxyribonucleic acid; FND, Fluorescent nanodiamonds.

#### Microfluidic Chip-Based POC Detection of DENV

Microfluidic platforms serve as compact POC diagnostic lab-on-a-chip that facilitates the manipulation of small fluid volumes through channels to perform complex analyzes.^71^ Recently, microfluidic platforms enabled the development of a 3D printed paper-based microfluidic POC prototype to detect dengue virus serotypes. The prototype incorporates both fluidic chip and paper components, utilizing polylactic acid (PLA) and wax filaments. PLA, a biodegradable thermoplastic material, is used for the 3D printing of fluidic chips because of its mechanical strength and suitability for precise structural design. Wax filaments are used to form hydrophobic barriers on the paper substrate. When heated, wax penetrates the paper matrix, creating hydrophobic regions that effectively confine fluid flow within specific channels. This design ensures precise fluid delivery, prevents cross-contamination between assay regions, and enhances the precision of diagnostic results. Hydrophobic barriers also facilitate capillary action, ensuring consistent and controlled fluid flow throughout the device for optimal performance. As illustrated in Figure 6a, these hydrophobic barriers are integral to the successful detection of dengue virus serotypes.^72^

**Figure 6:**
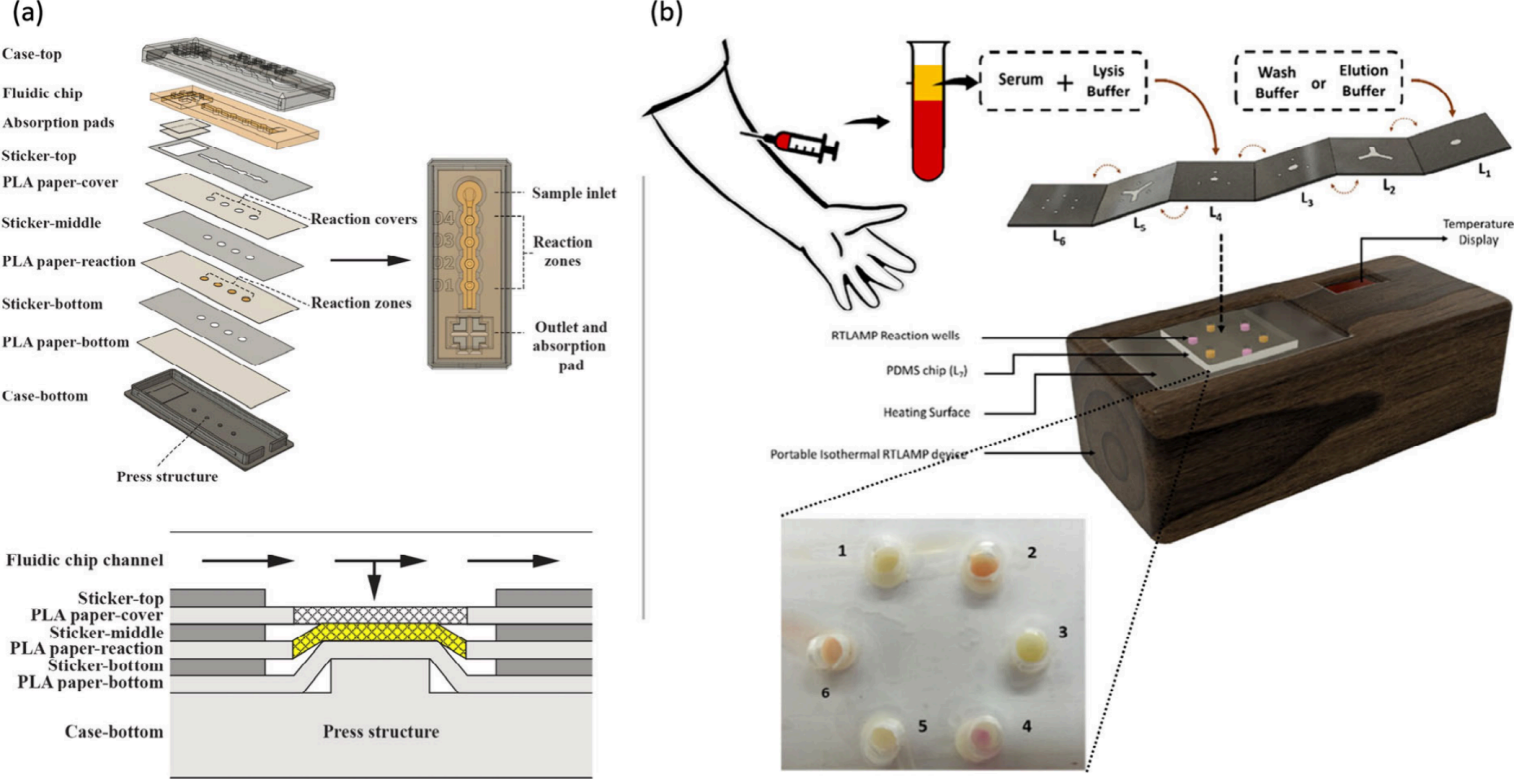
(a) Components of the fluid chip and paper of the μPAD, along with a cross-sectional view. Adapted with permission from ref.^72^ Copyright 2022 Permission from Elsevier. (b) Schematic illustration of the working principle of the origami-based microfluidic device, along with the visual representation of the test results in the current system. Adapted with permission from ref.^73^ Copyright 2022 Permission from the American Chemical Society.

A similar wax-patterned μPAD has been developed for the detection of dengue NS1 POC in human serum, achieving detection limits measured by the naked eye, a scanner, and a smartphone camera at 200 ng/mL, 46.7 ng/mL, and 74.8 ng/mL, respectively.^74^ These advances highlight the critical role of PLA and wax filaments in improving the operational efficiency and user-friendliness of POC diagnostic devices. Furthermore, the development of an all-in-one POC multiplex paper/polymer microfluidic device has been developed and utilizes a simple origami-based colorimetric detection method for all four serotypes of DENV.^73^ The working principle and visual output of the origami-based device are presented in Figure 6b. It was tested with 120 clinical samples, demonstrating an overall sensitivity of 97% and a specificity of 100%, with results obtained in approximately 30 min.

Another approach involves the use of cross-linked polymethacrylate microspheres integrated into a microfluidic disk for the rapid and sensitive detection of DENV in serum samples, achieving a detection limit of detection as low as 1.9 pfu/ml.^75^ A 3D microfluidic enzyme-linked immunosorbent assay (ELISA) platform has been developed, using a 3D stack surface modified with mouse anti-CD163 antibodies for the detection of soluble CD163 (sCD163) in serum samples, showing a high correlation with a R value of 0.9298 and a p-value of <0.0001.^76^ Additionally, a suction-type microfluidic immunosensing chip has been developed for the detection of DENV, achieving a sensitivity of as low as 101 PFU/mL.^77^ Furthermore, a microfluidic immunofluorescence platform has been developed using zinc oxide nanorods (ZnO NRs) functionalized with 4G2 monoclonal antibodies (mAbs) for the early detection of DENV-3 virus, demonstrating a detection range of 3.1 × 10^3^ ng/mL to 3.1 × 10^–4^ ng/mL.^78^ These compact microfluidic systems developed employed small volumes of fluids to perform diagnostic tests.

Table 2 provides a comprehensive comparison of various POC diagnosis platforms used for the detection of DENV biomarkers. Highlight detection methods, target biomarkers, sensitivity, and specificity, as well as the advantages and limitations of different POC diagnosis readout methods and portable POC devices for DENV diagnosis, including colorimetric assay, electrochemical method, lateral flow strip, and microfluidic chip platforms. Comparative analysis of electrochemical platforms versus other POC diagnosis methods reveals distinct advantages and limitations across various diagnostic tools. Electrochemical platforms, considered for high sensitivity and real-time detection, are better than colorimetric assays, lateral flow strips, and microfluidic chips in terms of sensitivity, with an LOD of as low as 0.16 nM for DENV biomarkers. These platforms provide precise quantitative measurements, offering a significant advantage over qualitative methods such as colorimetric assays. Although their sensitivity is high, electrochemical platforms face challenges such as high costs and the need for regular calibration^45^ which may limit their accessibility compared to the cost-effective and user-friendly lateral flow strips and colorimetric assays. On the contrary, microfluidic chips, although compact and require minimal sample volume, are complicated to fabricate, making their scalability and widespread use more challenging.^79^ Therefore, electrochemical platforms represent a promising option for advanced diagnostics, although their adoption is limited by factors such as cost and operational complexity.

**Table 2:** Comparison of Various POC Diagnosis Platforms for the Detection of DENV

POC diagnosis platforms	detection approachs	target biomarkers	sensitivity (%)	specificity (%)	limit of detection	advantages	limitations	ref
colorimetric assays	DNA/RNA hybridization and aggregation	NS1 antigen, DENV RNA	95	100	∼1 pg/μL	simple, rapid, and cost-effective	limited to qualitative analysis	^35,37^
electrochemical platforms	antigen–antibody interaction	DENV-NS1	90	80.95	1.63 pg/mL	high sensitivity and wider linear detection range	high cost and requires calibration	^31^
lateral flow strips	antigen–antibody interaction	DENV-NS1 and all serotypes	90.0, 88.24, 82.61, and 83.33	98.74, 96.13, 99.39, and 97.04	1.56 ng/mL	user-friendly and portable	lower sensitivity for some serotypes	^58,66^
microfluidic chips	origami-based microfluidic device and cross-linked polymethacrylate microfluidic disk	all DENV-NS1 serotypes and DENV serum samples	97	100	1.9 PFU/mL	compact, rapid, and requires minimal sample volume	fabrication complexity	^73,75^

## Predictive Biomarkers

Biomarkers are measurable indicators resulting from various biological materials, including human tissues, cells, and fluids.^80^ Biomarkers can be categorized into two primary subgroups: prognostic and predictive biomarkers.^81^ Prognostic biomarkers provide information on the natural course of a disease, independent of treatment, and help stratify patients based on their likely progression of the disease and the overall clinical outcome.^82–84^ In contrast, predictive biomarkers are used to determine the probability that a patient will respond to a specific therapeutic intervention, thus guiding treatment decisions and enabling personalized medicine approaches.^85^ While prognostic biomarkers assess disease severity and long-term results, predictive biomarkers help optimize therapeutic strategies by minimizing adverse effects and maximizing treatment efficacy.^86,87^

### Emerging Predictive Biomarkers for DENV

The identification of appropriate predictive biomarkers is essential for the early detection and management of severe dengue cases.^13^ These biomarkers allow for timely intervention by distinguishing between mild and severe forms of the disease, allowing healthcare workers to prioritize treatment and improve patient outcomes. This section explores emerging predictive markers, their clinical utility, and their potential for dengue diagnosis and prognosis. A recent study suggests that serological immune biomarkers, specifically antibody titers against immature viruses and recombinant E protein (rE proteins), can serve as predictors of disease severity in dengue.^88^ Antibody titers against the immature virus were significantly elevated in patients with severe dengue and dengue with warning signs (DWS) compared to those with dengue without warning signs (DWWS). However, antibody titers against rE proteins were higher in DWWS cases, suggesting that these antibodies may play a protective role. For immature viruses vs. rE protein, the use of single samples during hospital admission limits long-term evaluation, with findings not validated in all age groups or serotypes. Several studies have identified rapidly emerging predictive biomarkers classified by protein, nucleic acid, and metabolic biomarkers for severe dengue infection. Furthermore, the limitations identified for various dengue biomarkers highlight key challenges in their clinical utility and their applicability to research.

### Protein Biomarkers

Specifically, serum levels of lipopolysaccharide binding protein (LBP) and chymase have shown promise as early predictors of severe dengue.^89^ The study was carried out in the city of Asunción, Paraguay, from February 2018 to March 2020 (n = 145) and evaluated the associations of these biomarkers with the severity of the disease. The findings suggested that both LBP and chymase can serve as valuable tools for early detection and risk stratification in patients at risk of progressing to severe dengue. Similarly, serum ferritin levels have been investigated as an early predictive biomarker of the severity of dengue fever in adult patients with fever and thrombocytopenia.^90^ In a cohort of 350 patients with severe dengue, the median serum ferritin level was significantly higher at 3985 μg/L compared to 1936 μg/L in nonsevere cases, with a highly significant difference (*p* < 0.001) observed between the two groups. However, the detection of chymase and LBP faced limitations due to the small sample size and dependence on a single acute phase sample. Serum ferritin studies had no limitations.

### Nucleic Acid Biomarkers

Nucleic acid biomarkers are another promising approach to detect viral RNAs, such as zika, dengue, and chikungunya.^91^ Cell-free DNA (cfDNA) has been used as a predictive biomarker for the severity of dengue fever in the early acute phase. Dengue patients (n = 61) with shock syndrome (n = 8) had higher plasma levels of cfDNA. A cutoff value of >36.9 ng/mL predicted shock syndrome with good sensitivity (87.5%) and specificity (54.7%).^92^ Similarly, a study in Vietnam investigated the level of circulating cell-free DNA in acute dengue fever and found an increase in plasma levels.^93^ Furthermore, analysis of plasma samples from 39 patients with dengue identified miRNA signatures that serve as noninvasive predictive biomarkers for the early detection and monitoring of cases of severe dengue.^94^ A total of 89 miRNAs were detected, miR-486–5p, miR-92a-5p, miR-320a, miR-122–5p and miR-191–5p showed strong correlations with disease progression. In particular, miR-122–5p demonstrated high diagnostic precision, while miR-486–5p and miR-320a also showed significant potential to distinguish between different stages of dengue infection. Although miRNA expression analyzes were limited by small sample sizes and age-specific population.

### Metabolic Markers

Another study involving 110 patients identified aspartate aminotransferase (AST) and alanine aminotransferase (ALT) as an early predictive biomarker of dengue severity.^95^ AST showed a statistically significant negative correlation with platelet count (r = −0.23, p = 0.01) and a positive correlation with fluid accumulation (serositis) (r = 0.21, p = 0.02). This suggested that elevated AST levels may serve as an early indicator of severe dengue, correlated with thrombocytopenia and plasma leakage. Furthermore, soluble endoglin (ENG) and syndecan-1 (SDC-1), both vascular endothelial markers, have been identified as significant predictors of dengue disease outcomes and a predictive capacity with 100% precision (n = 47).^96^ A multicountry observational study reported that C-reactive protein (CRP) serves as a possible biomarker to predict disease progression in dengue.^97^ The study (n = 1120) identified that CRP levels of around 30 mg/L within the first 3 days of the illness were associated with the highest risk of severe dengue. Furthermore, nonstructural protein 1 (NS1) and immunoglobulin M (IgM) have demonstrated a high sensitivity in a predictive marker of the rapid POC diagnostic test for dengue infection, with a sensitivity of 95.4% for primary infections and 77.3% for secondary infections. The combination of the predictive marker of the NS1 antigen and the IgM antibody extended the diagnostic window, resulting in a general sensitivity of 89.4% and a specificity of 93.8%.^98^ Another study evaluated the correlation between average albumin levels and total urine protein using a colorimetric method to predict the severity of dengue fever.^99^ Furthermore, a comprehensive study of dengue fever highlights the development and validation of a set of 20 genes capable of predicting the progression to severe dengue with high precision.^100^ These reported predictive biomarkers are critical tools for the early identification of patients at risk of severe dengue, supporting more effective clinical decision making and resource prioritization.

Elevated AST/ALT levels were associated with severe illness, but did not provide gender-specific analysis. The ENG and SDC-1 studies did not specify any limitations. CRP demonstrated that limitations were minimized, although its association with fever and organ failure. NS1/IgM showed improved detectability in severe dengue cases. However, limited data on DENV 3 and DENV 4 serotypes and unassessed cross-reactivity with rickettsial and parasitic diseases were notable challenges. Urine protein/albumin levels were correlated with the severity of the disease, although limitations were not specified. Finally, the set of 20 genes as a molecular prognostic tool suffered from a limited validation sample size, indicating that more extensive investigation was needed to improve reliability in various situations.

### Emerging Predictive Biomarkers for Early Detection and Disease Progression in Severe DENV Infection

Several emerging predictive biomarkers for severe dengue have been identified, providing valuable information on early disease progression. Although some biomarkers, such as the NS1 antigen and C-reactive protein (CRP), enable early diagnosis, others, including chymase, LBP, and miRNA expression profiles, serve as indicators of disease severity at a later stage. Plasma cell-free DNA (cfDNA) levels have been found to be elevated in acute early dengue, making them a potential biomarker for early disease detection. Similarly, CRP levels within the first 3 days of illness have been associated with a higher risk of progression to severe dengue. On the contrary, biomarkers such as ENG and SDC-1 tend to increase toward the defervescence phase, making them more relevant for the assessment of later stage diseases.

### Key Findings and Limitations in Various Predictive Biomarkers in DENV Infection

The study of predictive biomarkers for DENV has achieved several important key findings, while also highlighting their limitations in diagnostic precision. Table 3 presents a comprehensive summary of the key findings and notable limitations identified in various studies on predictive biomarkers for the detection of DENV infection.

**Table 3:** Overview of Key Findings and Notable Limitations in Studies on Predictive Biomarkers for DENV^a^

biomarkers	key findings	limitations	early diagnosis of disease progression	ref
immature viruses vs rE protein	Immature virus antibody levels were significantly higher in DWS compared to DOS (*p* = 0.0006).	collection of single samples during hospital admission	no (more relevant for disease severity assessment)	^88^
		not valid in all age groups		
		not confirmed for all serotypes		
chymase and LBP	Elevated levels of anti-DENV IgG were detected in the pGOLD multiplex serologic assay.	small sample size	no (associated with later disease stages)	^89^
		only a single acute phase sample is available		
serum ferritin	DENV patients exhibited significantly higher median ferritin levels (3985 μg/l).		potentially (elevated early but requires further validation)	^90^
cell-free DNA	Plasma cfDNA levels predict severe dengue in acute illness.	cfDNA measurement is limited to patients with dengue, not other conditions.	yes (elevated in early disease phase)	^92,93^
miRNA expression	miR-122–5p demonstrated high diagnostic precision.	small sample size	potentially (requires further validation for early detection)	^94^
		study carried out only in patients in the adolescent to middle-aged groups		
AST/ALT	Individuals with elevated AST levels experienced more severe illness compared to those with normal levels.	small sample size	no (more relevant for liver dysfunction monitoring)	^95^
		does not account for gender predilection		
ENG and SDC-1	Increased levels of ENG and SDC-1 toward defervescence were observed.		no (more relevant for later-stage disease monitoring)	^96^
CRP	An association was observed between fever and organ failure (*P* = 0.011).	Limitations are minimized.	yes (elevated within the first 3 days of illness)	^97^
NS1/IgM	improved detectability in severe dengue cases	There is not enough data to assess the performance of the DENV 3 and DENV 4 serotypes.	yes (commonly used in rapid PoCT)	^98^
		rickettsial and parasitic diseases not evaluated		
urine protein/albumin levels	Higher urine protein/albumin levels were associated with more severe disease.		potentially (requires further validation for early detection)	^99^
20-gene set	identified as a potential molecular prognostic tool	limited validation sample size	no (requires further validation for early stage detection)	^100^

a AST, Aspartate Aminotransferase; ALT, Alanine Aminotransferase; cfDNA, Cell-free DNA; CRP, C-reactive protein; ENG, Endoglin; SDC-1, Syndecan-1; LBP, lipopolysaccharide binding protein; rE protein, recombinant E protein; NS1/IgM, nonstructural protein 1/Immunoglobulin M.

## Challenges and Limitations in POC Diagnosis of Severe DENV Infection

Although there have been significant advances in the diagnosis of POCs for severe dengue, several challenges remain. A major limitation is the varying sensitivity of POC diagnosis platforms in different dengue serotypes, which requires the development of universally effective diagnostic solutions. Dengue fever also overlaps significantly with similar infections of malaria, chikungunya, and the Zika virus, leading to frequent misdiagnosis. The presence of multiple serotypes further reduces the specificity of tests, particularly in terms of cross-reactivity between antibodies. Furthermore, it lacks precision to distinguish between primary and secondary dengue infections in terms of severity. In addition, reducing production costs and improving scalability for mass manufacturing are critical to ensuring widespread accessibility in resource-limited settings. Standardization of clinical testing procedures is also necessary to improve reliability and reproducibility in different healthcare settings. Addressing these challenges requires ongoing research, improved technological innovations, and the establishment of global standards to ensure that the diagnosis of severe dengue POC meets the demands of diverse clinical environments.

## Future Direction in POC Diagnosis of Severe DENV Infection

The future of point-of-care diagnosis for severe dengue requires disease-specific technological advancements that improve diagnostic precision, differentiate serotypes, and enable rapid clinical decision-making. Current limitations in dengue diagnostics, such as cross-reactivity with other flaviviruses, low sensitivity in early stage detection, and inability to predict disease progression, required targeted innovations.

One promising direction is the development of serotype-specific POC diagnosis platforms that can differentiate DENV-1 to DENV-4 infections with high sensitivity. This can be achieved through multiplexed POC biosensors that allow for simultaneous detection of multiple dengue serotypes.^101,102^ Such platforms offer high specificity, which is crucial in endemic regions where multiple serotypes cocirculate. Additionally, aptamer-based biosensors have shown high affinity and specificity for dengue viral antigens, providing an alternative to antibody-based detection systems.^103,104^ Recent studies show that RNA aptamers targeting the NS1 protein enhance diagnostic potential, making them suitable for low-cost paper-based POC diagnosis devices.^105^

Machine learning (ML) applications should also be refined to focus specifically on dengue prognosis rather than general fever diagnostics.^106,107^ Unlike broad artificial intelligence (AI) models, dengue-specific ML algorithms trained on platelet count trends, viral load variations, and inflammatory cytokine levels could predict which patients are at risk of developing severe dengue (DHF/DSS). This would enable early intervention and improved patient management. Another promising avenue is the use of nanomaterial-enhanced POC biosensors, particularly plasmonic and quantum dot-based assays, for the highly sensitive detection of early stage dengue infection.^108^ Plasmonic nanostructures functionalized with NS1-specific ligands could offer real-time detection with visual signal amplification, improving the accuracy of field-deployable POC diagnosis devices. Furthermore, portable POC LFA and microfluidic devices are evolving to provide user-friendly, low-cost, and rapid POC diagnosis tools.

Future research should focus on integrating these novel approaches into clinically validated, regulatory approved POC diagnosis platforms. Additionally, standardization of biomarker panels for the prognosis of dengue, such as the inclusion of soluble endoglin, syndecan-1, and miRNA signatures, could improve risk stratification in severe dengue cases. By focusing on dengue-specific technological innovations, POC diagnosis platforms can evolve beyond generic infectious disease diagnostics and serve as powerful tools for early detection, serotype differentiation, and disease prognosis.

## Summary and Conclusions

In this review, we have highlighted recent advances in POC diagnosis platforms and emerging predictive biomarkers that aim to improve the rapid diagnosis and management of severe DENV infection. POC diagnostic readout methods and portable POC devices for DENV diagnosis, including colorimetric assay, electrochemical method, lateral flow strip and microfluidic chip platforms, exhibit significant enhancements in diagnostic capabilities, particularly in resource-limited settings.

Aptamer-associated RT-LAMP colorimetric assays achieve remarkable diagnostic precision with 95% sensitivity and 100% specificity, providing reliable results even in early stages of infection. Similarly, simple antibody-AuNPs conjugated systems enable rapid visual detection of DENV, achieving detection limits as low as 1.56 ng/mL with simple antibody-conjugated systems. Advanced 3D printed paper-based microfluidic chips have achieved remarkable sensitivity, detecting DENV serotypes at concentrations as low as 1.9 pfu/mL. Electrochemical platforms further enhance diagnostic precision, with differential pulse voltammetric biosensors that demonstrate robust real-time analysis and ultralow detection limits of 1.63 pg/mL. Multiplexed approaches, such as the single test strip lateral flow platform using color mixing strategies and the disposable paper-based electrochemical genosensor, enable simultaneous detection of all four dengue serotypes, ensuring comprehensive diagnostics. In addition, integrating POC diagnosis devices with smartphone systems improves diagnostic accessibility and real-time monitoring, bridging the gap between technological innovation and practical application.

Emerging key predictive biomarkers, such as lipopolysaccharide binding protein and chymase, have shown utility in predicting the severity of the disease. Additionally, serum ferritin levels have been identified as a reliable early marker. Particularly elevated ASTs are associated with thrombocytopenia and plasma leakage, making them useful indicators of severe dengue. Vascular endothelial markers, including soluble endoglin and syndecan-1, offer 100% predictive accuracy of disease severity, while specific miRNA signatures, such as miR-122–5p and miR-486–5p, provide noninvasive predictive capabilities, closely correlated with disease progression. C-reactive protein (CRP) and NS1 antigen, combined with IgM antibody detection, have also been highlighted for their diagnostic value, extending the diagnostic window and improving sensitivity.

In conclusion, the rapid evolution of portable POC diagnostic technologies and the identification of emerging predictive biomarkers have significantly advanced the diagnosis and treatment of severe viral infection. of dengue. POC diagnosis platforms such as colorimetric assay, lateral flow strip, microfluidic chip, and electrochemical biosensors have shown remarkable sensitivity, specificity, and ease of use, providing critical diagnostic capabilities at the bedside, particularly in resource-limited settings. At the same time, emerging predictive biomarkers, including lipopolysaccharide-binding protein, chymase, serum ferritin, and miRNA signatures, offer valuable information on disease progression, facilitating early detection of severe cases. Integration of these innovative diagnostic approaches has the potential to revolutionize dengue management, allowing for timely clinical intervention and reducing morbidity and mortality.

The integration of predictive biomarkers, such as protein, nucleic acid, and metabolic biomarkers, into POC diagnosis platforms has great potential to revolutionize dengue diagnostics. These biomarkers not only provide an indicator of sensitivity and specificity but also provide mechanistic understandings of disease progression, enabling timely and accurate patient management. Standardized statistical reporting and structured biomarker categorization will further enhance their clinical utility and research applicability. However, challenges such as viral serotype diversity, the need for standardized protocols, and technological scalability remain. Addressing these challenges through continued research and innovation is essential to realize the full potential of POC diagnostic technologies and predictive biomarkers to improve global dengue outcomes.

## ABBREVIATIONS

AI: Artificial Intelligence
ALT: Alanine Aminotransferase
ANN: Artificial Neural Network
APTA-RT-LAMP: Aptamer-associated reverse transcription loop-mediated isothermal amplification
AST: Aspartate Aminotransferase
AuNPs: Gold nanoparticles
AuNSP@mAb: Gold nanospheres and monoclonal antibody
BSA: Bovine serum albumin
CBC: Complete blood count
CHIKV: Chikungunya virus
CISS: Chiral-induced spin selectivity
CRP: C-reactive protein
CSF: Coronary slow-flow
CVD: Cardiovascular diseases
DENV: Dengue virus
DENV5: Dengue virus fifth serotype
DHF: Dengue hemorrhagic fever
DNA: Deoxyribonucleic acid
DWWS: Dengue without warning signs
DPV: Differential pulse voltametric
DSS: Dengue shock syndrome
DWS: Dengue with warning signs
EDC/s-NHS: 1-ethyl-3-(3-dimethylaminopropyl)­carbodiimide hydrochloride/N-hydroxysulfosuccinimide
ENG: Endoglin
Fe_2_O_3_@AuNPs: Iron oxide and gold nanoparticles
Fe_3_O_4_ NPs: Iron oxide nanoparticles: GCE, Glassy carbon electrode
GO-SiO_2_: Graphene oxide-silicon dioxide
IgG: Immunoglobulin G
IgM/IgG: Immunoglobulin M and Immunoglobulin G
LBP: Lipopolysaccharide-binding protein
LFA: Lateral flow assay
LFB: Lateral flow biosensor
LFBs: Lateral flow biosensors
LFD: Lateral flow dipstick
LFIA: Lateral flow immunoassay
LOD: Limit of detection
miRNA: MicroRNA
ML: Machine Learning
NS1: Nonstructural protein-1
PAD: Paper-based Analytical Device
PCR: Polymerase chain reaction
PDA: Polydopamine
PFU: Plaque forming unit
PLA: Polylactic acid
PMNs: Plasmonic magnetic nanoparticles
POC: Point-of-care
PoCT: Point-of-care testing
poly­(A) ssDNA: Poly­(A) single-stranded DNA
PPT-RTcPCR: Plasmonic photothermal reverse transcription colorimetric polymerase chain reaction
R^2^: Correlation coefficient RDT, Rapid diagnostic test
RNA: Ribonucleic acid
RT-LAMP: rE-protein, Recombinant E proteins
Reverse: transcription loop-mediated isothermal amplification
RT-RAA: Reverse transcription recombinase-aided amplification
sCD163: Soluble CD163
SDC-1: Syndecan-1
SELFIA: Spin-enhanced lateral flow immunoassay
SPCEs: Screen-printed carbon electrodes
SPEs: Screen-printed electrodes
TAg NPs: Triangular silver nanoparticles
ZnO NRs: Zinc oxide nanorods
μg/mL: Microgram per milliliter
μPAD: Microfluidic paper-based analytical device
